# Effects of Nitrate Supplementation on Exercise Performance in Humans: A Narrative Review

**DOI:** 10.3390/nu13093183

**Published:** 2021-09-13

**Authors:** Matjaž Macuh, Bojan Knap

**Affiliations:** 1 Department of Food Science and Technology, Biotechnical Faculty, University of Ljubljana; Jamnikarjeva 10, 1000 Ljubljana, Slovenia; 2 Department of Nephrology, University Medical Centre Ljubljana, Zaloška 7, 1000 Ljubljana, Slovenia; knap.j.bojan@gmail.com; 3 Faculty of Medicine, University of Ljubljana, Korytkova ulica 2, 1000 Ljubljana, Slovenia

**Keywords:** nitrate, sports, performance, nitric oxide, dietary supplements, exercise science

## Abstract

Nitrates have become increasingly popular for their potential role as an ergogenic aid. The purpose of this article was to review the current scientific evidence of nitrate supplementation on human performance. The current recommendation of nitrate supplementation is discussed, as well as possible health complications associated with nitrate intake for athletes, and dietary strategies of covering nitrate needs through sufficient intake of nitrate-rich foods alone are presented. Pubmed, Scopus, and Web of Science were searched for articles on the effects of nitrate supplementation in humans. Nitrates are an effective ergogenic aid when taken acutely or chronically in the range of ~5–16.8 mmol (~300–1041 mg) 2–3 h before exercise and primarily in the case of exercise duration of ~10–17 min in less trained individuals (VO_2max_ < 65 mL/kg/min). Nitrate needs are most likely meet by ingesting approximately 250–500 g of leafy and root vegetables per day; however, dietary supplements might represent a more convenient and accurate way of covering an athlete’s nitrate needs. Athletes should refrain from mouthwash usage when nitrate supplementation benefits are desired. Future research should focus on the potential beneficial effects of nitrate supplementation on brain function, possible negative impacts of chronic nitrate supplementation through different nitrate sources, and the effectiveness of nitrate supplementation on strength and high-intensity intermittent exercise.

## 1. Introduction

Nitric oxide (NO) is a signaling molecule involved in numerous vascular and cellular functions, such as cellular respiration, vasodilation, and angiogenesis. NO is produced through both endogenous and exogenous pathways by dietary nitrate (NO_3_^−^) ingestion. NO_3_^−^ is an active compound found in certain NO_3_^−^ rich vegetables and elicits potentially ergogenic as well as health-promoting effects. As such, NO_3_^−^ presents as an interesting compound from both a sports perspective as an ergogenic aid as well as a potentially cost-effective tool for reducing the likelihood of cardiovascular events [1]. 

NO impacts exercise performance through several mechanisms: decreased fatigue during exercise, increased nutrient and oxygen delivery to the working muscles, and increased excretion of metabolic by-products of high-intensity exercise. There are several supplements promoted as so-called ‘NO boosters’. Historically, the amino acid L-arginine has been used extensively in these products to increase blood flow through NO production. Later, the non-essential amino-acid L-citrulline, found primarily in watermelons, cucumbers, and other melons, has replaced L-arginine in these supplements to a certain degree. Oral intake of L-citrulline as a dietary supplement increases the bioavailability of L-arginine to a greater extent than supplementation with L-arginine, as it is directly transported to the kidneys where it is converted to L-arginine, whereas L-arginine is subjected to catabolism via the enzyme arginase [2]. An extensive review of the literature on the effects of L-arginine and L-citrulline is beyond the scope of this article. However, it is worth noting that the positive effects of increased NO bioavailability may be induced through not only NO_3_^−^ supplementation but potentially through L-arginine and L-citrulline supplementation as well.

NO_3_^−^ supplementation and its effects on different types of exercise performance have gained attention in the past 2 decades. The purpose of this narrative review was to review the current scientific literature on the effects of NO_3_^−^ supplementation on human performance, mechanisms by which NO_3_^−^ may act as an ergogenic aid, and whether NO_3_^−^ supplements are needed. The review was conducted using the online databases PubMed, Scopus, and Web of Science by searching keywords of ‘nitrate supplementation’ and ‘nitrate supplementation exercise performance’. Studies published up to September 2021 (English language restriction) were included. Studies completed in animal models or individuals with certain medical conditions were excluded from the review.

### 1.1. Nitrates: Mechanisms of Action as an Ergogenic Aid

NO_3_^−^ may improve sports performance primarily through its effects on skeletal muscle, blood vessels, and the brain [3]. Increased NO availability can affect mitochondrial respiration and biogenesis, increase blood flow in active muscles during physical activity, and consequently reduce adenosine triphosphate (ATP) consumption during muscle contraction, and reduce oxygen consumption during aerobic exercise [4]. The effect of NO_3_^−^ on blood vessels is manifested through blood pressure reduction, an observation also seen in otherwise healthy populations with blood pressure values in reference ranges. NO_3_^−^ may also increase heat loss through the skin during physical activity [3]. Research on the effect of NO_3_^−^ on the brain is currently in its infancy and is not as well understood as are the effects on skeletal muscles and blood vessels. As such, there is currently no direct evidence that the addition of NO_3_^−^ could, in fact, increase the availability of NO in the brain. Nevertheless, research completed in animal models reports that NO in the brain reduces oxygen consumption during exercise [5], accelerates heat loss through the skin during physical activity [6], and potentially exhibits protective effects against exercise-induced hyperthermia [6,7]. 

### 1.2. Nitrates: Metabolism

NO is synthesized either by ingesting foods rich in NO_3_^−^, L-arginine, L-citrulline, or through endogenous synthesis. NO synthesis from L-arginine takes place via its oxidation, catalyzed by the family of enzymes called nitric oxide synthase (NOS), and takes place in the presence of oxygen. The oxidation of L-arginine to NO was once thought to be the only way NO is formed in the body. However, we now know that this is not the case and that NO can be produced through the reduction of NO_3_^−^ and nitrites (NO_2_^−^) in the body [8]. NO formation via the NO_3_^−^–NO_2_^−^–NO pathway takes place with a gradual decrease in oxygen concentration (hypoxia), which occurs during intense physical activity. This pathway can be described as an alternative or complementary pathway of NO formation in the absence of oxygen to the aforementioned pathway via the amino acid L-arginine [9].

After ingestion of NO_3_^−^ from diet or dietary supplements, plasma NO_3_^−^ levels peak after approximately 1–2 h or after 2–3 h for NO_2_^−^ before concentrations of both compounds gradually decrease and return to baseline levels after approximately 24 h [10].

Digestion of ingested NO_3_^−^ and NO_2_^−^ begins in the mouth, where a certain portion (~25%) of ingested NO_3_^−^ is digested by saliva via anaerobic bacteria that reside there and reduce NO_3_^−^ to NO_2_^−^. The greater part of NO_3_^−^ reduction takes place later in the stomach because of the low pH of the environment. Further reduction of NO_2_^−^ takes place via a non-enzymatic reaction in the gastric lumen. Most of the circulating NO_3_^−^ is eventually excreted through urine. However, approximately 20–25% of NO_3_^−^ is taken up by the pancreas from the bloodstream and concentrated in saliva. The cycle of NO_3_^−^ metabolism is thus completed and is then repeated, where the anaerobic bacteria in human saliva initially reduce NO_3_^−^ to NO_2_^−^. NO_2_^−^ and remaining non-reduced NO_3_^−^ is then swallowed again where a smaller percentage of NO_2_^−^ is further reduced to NO by the low pH environment of the gastric lumen [9]. Most of the remaining NO_2_^−^, however, re-enters the systemic circulation and is transported to specific locations throughout the body where they are reduced to NO via various enzymatic or non-enzymatic degradation pathways. The exact mechanism of NO_2_^−^ re-entry into the circulation is unknown, but the reduction takes place primarily in the vascular system under conditions of hypoxia and reduced pH levels. Through the NO_3_^−^–NO_2_^−^–NO pathway of NO_3_^−^ degradation, endogenously ingested nitrates are recycled by oral bacteria and act as a kind of reservoir of NO synthesis precursors [11].

It is important to note that this NO_3_^−^ recycling process is severely impaired when oral mouthwashes are used on a regular basis as they destroy oral bacteria and consequently lower plasma NO_3_^−^ levels [12,13]. This may be an important implication for athletes who should possibly be cautioned against mouthwash usage, especially during NO_3_^−^ supplementation periods. Interestingly, chlorine-sterilized pool water does not seem to impair the NO_3_^−^ reduction pathway through oral bacteria breakdown, as previously speculated by some. This has important implications for swimmers, a population where NO_3_^−^ supplementation might be efficacious [14].

## 2. Nitrates and Exercise Performance

NO_3_^−^ have been frequently researched over the past decade and a half in terms of their impact on sports performance in a wide variety of training modalities. One of the first of such studies reported positive effects of NO_3_^−^ supplementation on time to exhaustion [15]. Afterward, NO_3_^−^ supplementation was found to have a positive impact on lowering oxygen consumption and time to exhaustion [16]. 

Several other studies report positive benefits of NO_3_^−^ intake, such as lowering blood pressure [17,18], reduced use of ATP and reduced degradation of phosphocreatine (PCr), improved muscle contractile efficiency [16], reduced oxygen consumption during submaximal exercise [19,20,21], and improved performance [16,19,22,23,24]. Conversely, many other studies do not report the above-mentioned positive effects [25,26,27,28,29,30,31]. A more detailed analysis of studies examining the effects of NO_3_^−^ supplementations on exercise performance is presented in Table 1.

The observation that NO_3_^−^ supplementation effects are less pronounced in better-trained individuals is also supported in a systematic review and meta-analysis by Campos et al. (2018) [3]. These researchers reported that despite the smaller impact of NO_3_^−^ on the performance of well-trained individuals, the effect of NO_3_^−^ should not be neglected. NO_3_^−^ is currently regarded as one of a handful of dietary supplements with a direct, positive effect on athlete’s performance based on the latest consensus statement by the International Olympic Committee (IOC) [72]. The effect of NO_3_^−^ supplementation on performance may be particularly desirable on competition day, where the differences between the competitors are marginal.

Additionally, diet may also influence NO_3_^−^ supplementation effects in well-trained individuals. This effect, speculative in nature, relates to the general eating habits of elite athletes. We can assume that most top athletes include decent amounts of foods rich in NO_3_^−^ as well as L-arginine and L-citrulline, making the effect of the dietary supplementation less pronounced [4]. Of course, the latter works solely on the assumption that the athlete consumes enough NO_3_^−^-rich foods, and if the intake of NO_3_^−^ from the diet is too low, dietary supplement usage will most likely yield greater benefits. A parallel may be drawn with creatine supplementation. Vegetarians and vegans who consume diets poorest in creatine (meat, fish, and eggs) have the lowest levels of muscle creatine phosphate, and the effect of creatine supplementation on performance is significantly more pronounced in this population versus omnivores [73].

Lastly, a difference in performance outcomes observed in studies using NO supplementation might be attributed to NO’s ability to interact with other free radicals. As NO half-life in vivo is in the order of a few seconds, this reaction might be dependent on its initial concentration [74], thus making it potentially problematic in the context of NO supplementation where a bolus of NO_3_^−^ is ingested at once without the presence of other ingredients affecting its digestion. In this context, a major concern might be the interaction between NO and superoxide (O_2_) leading to peroxynitrite (OONO^−^) formation—a highly reactive nitrogen species (RNS) affecting mitochondrial function, signal transduction, and stress response [75,76]. Chronic OONO^−^ formation might directly lead to the production of RNS and reactive oxygen species (ROS) in other subcellular compartments, leading to increased oxidative stress [77], which might affect performance. As with other supplements affecting oxidative stress, special emphasis on using such supplements in the right context might have to be taken when using NO_3_^−^ supplementation. For example, high doses of vitamin C and E have been shown to blunt aerobic exercise adaptations [78,79]. High antioxidant usage has also been shown to blunt body composition improvements following a resistance training protocol [80]. This might limit the usage of such supplements during specific conditions where an athlete’s recovery is more important than optimal adaptation (e.g., tournaments where an athlete has many competitions in a short time frame). For NO_3_^−^ supplementation, no such guidelines can be given, and to our knowledge, no research has looked directly into differences in RNS formation from NO_3_^−^ supplementation or via NO_3_^−^ rich diet but should be taken into account, especially when chronic NO_3_^−^ supplementation is being considered.

There are also several literature reviews and meta-analyses examining the effectiveness of NO_3_^−^ supplementation on exercise performance. Hoon et al. (2013) reported a statistically significant improvement in performance for constant power/speed tests and smaller, statistically insignificant but positive effects in the case of incremental tests and time trials [81]. However, the meta-analysis did not consider differences in NO_3_^−^ supplementation protocols between trials as well as the training status of individuals. Pawlak-Chaouch et al. published a meta-analysis including 26 randomized and placebo-controlled studies in 2016 and reported a significant reduction in VO_2_ during submaximal exercise [82]. Afterward, a meta-analysis by Van De Wall and Vukovich in 2018 reported that NO_3_^−^ supplementation can improve tolerance to and efficiency of continuous high-intensity exercise and maximal exercise with increasing intensity [4]. The authors recommend the effectiveness of both acute as well as chronic NO_3_^−^ supplementation (up to 15 days) when taken in an amount of 5–9 mmol with similar conclusions being found in a meta-analysis by McMahon et al. (2016) on the impact of NO_3_^−^ supplementation specifically on endurance performance [83]. This meta-analysis included 47 studies and reported that the effect of NO_3_^−^ supplementation was efficient on submaximal aerobic capacity, but lower effectiveness for time trial tests, which is in line with past research by Hoon et al. (2013) [81].

### 2.1. Nitrates: Supplementation Protocol

Even though a linear correlation exists between the amount of NO_3_^−^ ingested and the increase in plasma NO_3_^−^ levels, we can assume that there is an upper limit of NO_3_^−^ intake that still elicits a positive effect on performance. Wylie et al. (2013) reported that ingestion of 4.2 mmol NO_3_^−^ did not affect VO_2_ during moderate-intensity cycling, but VO_2_ was affected by NO_3_^−^ supplemented at 8.4 mmol and 16.8 mmol [39]. However, ingestion of 16.8 mmol NO_3_^−^ compared to 8.4 mmol NO_3_^−^ did not provide additional benefits. Thus, we can assume that the effect of NO_3_^−^ on performance exists within a specific interval. This interval is most likely between 5–9 mmol (310–560 mg) NO_3_^−^ taken either acutely 2–3 h before exercise [41] or chronically over an extended period [4]. Similar recommendations can also be found from the IOC [72] and Senefeld et al. (2020) [84]. The authors of the latter meta-analysis report that the effect of NO_3_^−^ is not statistically significant if NO_3_^−^ is taken less than 2 h before exercise as this does not allow enough time for NO_3_^−^ to NO conversion.

The effect of acute or chronic NO_3_^−^ intake is expected to be similar based on the current literature [84]; however, chronic NO_3_^−^ intake of more than 3 consecutive days before the race may potentially reap greater benefits for well-trained athletes [85].

### 2.2. Nitrates: Effects of Exercise Type and Conditions

The IOC reports the effectiveness of NO_3_^−^ differs across not only training status but exercise type and trial duration as well [72]. As such, the impact of NO_3_^−^ supplementation is reported to be in the range of 4–25% for time to exhaustion tests and 1–3 % for sport-specific tests lasting less than 40 min. NO_3_^−^ is expected to have the greatest effect between the range of approximately 12 and 40 min. Furthermore, within this time frame, the effects of NO_3_^−^ supplementation are likely most pronounced for exercise lasting between 601 and 999 s (~10–17 min), with the effects of NO_3_^−^ being effective regardless of normoxic or hypoxic conditions [84].

Effects of NO_3_^−^ supplementation have been studied in a wide variety of performance tests. However, the effects are most likely especially pronounced in time to exhaustion tests rather than time trial tests or incremental power tests. This may be due to the fact that time to exhaustion tests are supposedly better at measuring an athlete’s endurance capacity and are highly influenced by psychological factors (e.g., motivation, boredom, etc.) [3,86]. As for the type of exercise, Senefeld et al. (2020) report a significant effect of NO_3_^−^ on cycling and running, the most commonly studied training modalities in research on NO_3_^−^ supplementation, but not in knee extension tests or rowing rests. However, the lack of effect is most likely due to the relatively low proportion of studies completed on these two forms of performance tests rather than the exercise type per se [84].

NO_3_^−^ might be particularly effective for team sports athletes because of their potential beneficial effect on cognition. Athletes who participate in team sports are forced to make many quick decisions during training and competition. However, prolonged high-intensity exercise can have a negative impact on reaction time and task performance [87]. Thompson et al. (2015) reported a statistically significantly shorter reaction time in individuals receiving NO_3_^−^ supplementation in the amount of 6.4 mmol to 12.8 mmol for 7 consecutive days [88]. These positive effects of NO_3_^−^ supplementation on cognition may arise from the positive effect of NO on neurovascular coupling [89] and increased cerebral perfusion, primarily in the prefrontal cortex responsible for executive function [90]. NO_3_^−^ thus has a potentially positive effect on reducing the decline in cognitive function, primarily athlete’s reaction time, which is otherwise associated with repetitive high-intensity intermittent exercise.

A large majority of studies on NO_3_^−^ supplementation effects on performance have been completed on endurance tests. Some research, however, focuses on investigating these effects on high-intensity exercise and strength, where mixed results are observed. Thompson et al. (2016) reported improvements in sprints in the Yo-Yo test after NO_3_^−^ supplementation [91]. A similar effect in the same test in a sample of 32 football players is also reported by Nyakayiru et al. (2017) [24]. Cuenca et al. (2018) also reported an ergogenic effect of acute NO_3_^−^ intake of 6.4 mmol in the Wingate test, primarily in the first half of the sprints [50]. However, Martin et al. (2014) do not report a positive effect of NO_3_^−^ on the protocol of 8 s sprints with 30 s pauses [43].

A systematic review by San Juan et al. (2020) on the effect of NO_3_^−^ on weight training in an otherwise limited sample of four studies reported a positive effect of NO_3_^−^ on upper body strength and the number of repetitions performed in upper body strength test (bench press) as well as lower body strength test (squat) [92]. We certainly need more research into the impact of NO_3_^−^ on high-intensity exercise and strength, but preliminary results suggest that NO_3_^−^ could be beneficial in this sport context as well.

Another avenue of NO_3_^−^ effects on performance is research completed in extreme conditions, such as hypoxic and cold environmental settings (e.g., mountaineering, skiing, altitude training, etc.). As altitude increases, hypoxic conditions reduce O_2_ availability and decrease exercise performance. We can somewhat overcome this problem with altitude acclimatization; however, this process may take up to several weeks to fully manifest, which is not always possible in certain sports situations. Additionally, physical fitness otherwise seen at sea level might not ever be fully regained, even with prolonged acclimatization [93]. It is suggested that NO plays an essential role in hypoxia-induced vasodilatation, thereby ensuring adequate O_2_ availability to the working muscle and brain tissue during hypoxic conditions [94,95]. Certain populations native to higher altitudes (e.g., Sherpa) have been proposed to exert abnormal hypoxic tolerance in part due to elevated circulating levels of NO [96]. Indeed, research completed at simulated altitude shows the benefits of NO_3_^−^ supplementation on certain physiological parameters (e.g., improved mitochondrial respiration, O_2_ consumption during exercise, etc.). However, these findings do not seem to be observed in field tests at ’real’ altitudes, making real-world applications limited [93,96,97]. Certain researchers have postulated that chronic NO_3_^−^ supplementation might even be detrimental for athletes training at altitude from a perspective of possibly blunting hypoxic adaptations by decreasing arterial and muscle O2 saturation, which may act as a signal for such adaptations [98]. As such, there is currently no clear benefit of NO3- supplementation for athletes performing at high altitudes, and more research is needed on this specific topic.

### 2.3. Nitrates: Food Sources and Supplementation

The primary sources of NO_3_^−^ and NO_2_^−^ is either through NO_3_^−^ rich foods or through endogenous productions. Of these pathways, nutrition represents the one with greater potential to supply the body with a higher amount of NO_3_^−^ as the endogenous supply of NO_3_^−^ is relatively limited, and only a bowl of green leafy vegetables contains a higher amount of NO_3_^−^ than is formed endogenously throughout the entire day [9]. Athletes should thus be advised to meet their NO_3_^−^ through nutrition, either with NO_3_^−^ supplementation or through NO_3_^−^ rich foods—primarily leafy greens and root vegetables.

However, the NO_3_^−^ content of these vegetables varies greatly, as it depends on many factors such as the origin of the vegetable, the quality and pH of the soil in which the vegetables are grown, type and frequency of nitrogen fertilizers, type of vegetable cultivation, time of vegetable harvesting, age of the plant at harvest, conditions of vegetable storage and weather conditions in which vegetables are grown, and method of vegetable preparation, etc. [99].

Given all these factors, it is difficult to make a specific recommendation for athletes to meet the needs of NO_3_^−^ via the diet due to the large number of variable factors that affect the NO_3_^−^ content in the diet. Speculations can be made based on current data on the average NO_3_^−^ of NO_3_^−^ rich foods (e.g., beetroot, endive, fennel, kohlrabi, lettuce, pak choi, radish, rocket, and spinach) that this figure would be set at 150 g of aforementioned foods at the lowest [100]. However, this number might be significantly higher or possibly lower in some cases, depending on the above-mentioned factors. As a higher vegetable intake than 150 g is generally recommended, athletes should probably be encouraged to ingest approximately 250–500 g of leafy and root vegetables per day to ensure adequate NO_3_^−^ intake.

Additionally, ingesting a bolus of NO_3_^−^ via supplementation might hold a greater risk of peroxynitrite production relative to covering NO_3_^−^ needs through diet. This might be another limiting factor of NO_3_^−^ supplementation, as discussed in the chapter titled ’Nitrates and exercise performance’.

Lastly, NO_3_^−^ in the form of a dietary supplement may represent a more convenient and accurate way to cover the needs for NO_3_^−^; however, as with any other dietary supplement, there is always the possibility of supplement contamination [101], and an athlete’s budget must also be considered.

## 3. Conclusions

Based on current literature, NO_3_^−^ represents an effective ergogenic aid for improving performance through various mechanisms and is useful in a variety of sports situations and exercise modalities. The effect of NO_3_^−^ is most pronounced in less-trained individuals when taken acutely or chronically in the range of ~5–16.8 mmol (~300–1041 mg NO_3_^−^) 2–3 h before exercise and primarily in the case of exercise duration of ~10–17 min. Nitrate supplementation is less pronounced in well-trained individuals (VO_2max_ > 65 mL/kg/min); however, it might still be desirable, especially during competition. Athletes should refrain from mouthwash usage when nitrate supplementation benefits are desired.

NO_3_^−^ is found in certain vegetables, but due to many variable factors, we cannot make exact recommendations to cover these needs through diet alone. Given that there is a potential for supplement contamination, it would make sense to explore how dietary needs for NO_3_^−^ can be covered through dietary sources. Currently, speculations can be made that this figure is roughly 250–500 g of leafy and root vegetables per day. Dietary supplements might represent a more convenient and accurate way of covering one’s needs for nitrate; however, potential supplement contamination and an athlete’s budget must be considered.

Future research should focus on the potential beneficial effects of NO_3_^−^ on the brain, especially in regard to sport-specific situations, and on the effectiveness of NO_3_^−^ in strength training and high-intensity intermittent training.

## Figures and Tables

**Table 1:** Effects of nitrate supplementation on exercise performance.

Study (Year)	Number of Participants (Sex)	Participant Characteristics	Supplementation Protocol	Performance Protocol (Measured Variable)	Main Findings
Larsen et al. (2007) [15]	9 (7 M, 2 F)	Cyclists and triathlon competitors (VO_2peak_ 55 ± 3.7 mL/kg/min)	0.033 mmol NO_3_^−^/kg BM for 2 consecutive days thrice daily	Incremental ergometer test (time in s)	↔ Time to exhaustion
Bailey et al. (2009) [16]	8 (M)	Healthy and recreationally active VO_2max_ 49 ± 5 mL/kg/min)	5.5 mmol NO_3_^−^ for 6 consecutive days	High-intensity exercise (time in s)	↓ O_2_ uptake during high-intensity exercise ↑ Time to exhaustion ↓ Systolic blood pressure
Bailey et al. (2010) [17]	7 (M)	Healthy and recreationally active	5.1 mmol NO_3_^−^ for 6 consecutive days	High-intensity exercise (time in s)	↓ Muscle phosphocreatine degradation ↑ Time to exhaustion ↑ ATP turnover
Vanhatalo et al. (2010) [18]	8 (5 M, 3 F)	Healthy individuals	5.2 mmol NO_3_^−^ twice daily for 15 consecutive days	Incremental cycling test (power in W)	↓ Steady-state VO_2_ ↑ Peak power and work rate
Larsen et al. (2010) [32]	9 (7 M, 2 F)	Healthy and recreationally active (VO_2max_ 3.72 ± 0.33 mL/kg/min)	0.1 mmol NO_3_^−^/kg BM for 2 consecutive days	Incremental ergometer test (time in s)	↔ Time to exhaustion
Vanhatalo et al. (2011) [33]	9 (7 M, 2 F)	Healthy and recreationally active	9.3 mmol NO_3_^−^ split into three doses taken 24, 12, and 2.5 h prior to testing	Knee extension (time in s)	↑ Knee extension performance ↑ PCr recovery time constant
Lansley et al. (2011) [22]	9 (M)	Well-trained cyclists VO_2peak_ 56 ± 5.7 mL/kg/min)	≃6.2 mmol NO_3_^−^	4 km and 16.1 km time trial (time in min and power in W)	↑ Power output in both 4 km and 16.1 km trial ↑ Performance in both 4 km and 16.1 km trial
Murphy et al. (2011) [23]	11 (5 M, 6 F)	Healthy and recreationally active	500 g beetroot (≃500 mg or 8 mmol NO_3_^−^)	5 km running time trial (velocity in km/h)	↔ Performance ↓ RPE
Masschelein et al. (2012) [19]	15 (M)	Healthy and recreationally active (VO_2peak_ 61.7± 2.1 mL/kg/min)	0.07 mmol NO_3_^−^/kg BM/day for 6 consecutive days	Incremental ergometer test (time in s)	↓ VO_2_ and ↑ arterial O_2_ saturation during rest and exercise in hypoxic conditions
Bescos et al. (2012) [34]	13 (M)	Cyclists and triathlon competitors	11.8 mmol NO_3_^−^	Incremental test (time in s and power in W)	↔ Mean distance ↔ Power output
Peacock et al. (2012) [30]	10 (M)	Cross-country skiers (VO_2max_ 69.6 ± 5.1 mL/kg/min)	1 g KNO_3_ (9.9 mmol or 614 mg NO_3_^−^)	5 km running time trial (time in s)	↔ Time trail performance ↔ O_2_ cost
Bond et al. (2012) [35]	14 (M)	Rowers	5 mmol NO_3_^−^ for 6 consecutive days	6 × 500 m ergometer test at high-intensity (time in s)	↔ Rowing performance
Cermak et al. (2012) [36]	12 (M)	Cyclists and triathlon competitors (VO_2peak_ = 58 ± 2 mL/kg/min; W_max_ = 342 ± 10 W)	8 mmol NO_3_^−^ for 6 consecutive days	10 km running time trial (time in s and power in W)	↑ Time trial performance ↑ Power output
Cermak et al. (2012) [25]	20 (M)	Cyclists and triathlon competitors (VO_2peak_ 60 ± 1 mL/kg/min; W_max_ 398 ± 7.7 W)	8.7 mmol NO_3_^−^	Cycling at 75 % W_max_ to ≃1073 kJ (caloric-expenditure-based time trial) (time in min and power in W)	↔ Time trial ↔ Power output ↔ HR
Kelly et al. (2013) [37]	9 (M)	Healthy and recreationally active (VO_2max_ 54.5 ± 7.5 mL/kg/min)	8.2 mmol NO_3_^−^ for 5 consecutive days	Cyclic ergometry at 1) 60%, 2) 70%, 3) 80%, and 4) 100% W_max_ (time in s)	↑ Exercise tolerance at 60%, 70%, and 80% peak power ↔ At 100% peak power
Breese et al. (2013) [38]	9 (4 M, 5 F)	Healthy individuals	8 mmol NO_3_^−^ for 6 consecutive days	Incremental cycling test (time in s)	↑ VO_2_ kinetics ↑ Time-to-task failure
Wylie et al. (2013) [39]	10 (M)	Healthy individuals	(1) 4.2 or (2) 8.4 or (3) 16.8 mmol NO_3_^−^	Cycling to complete exhaustion (time in s)	↓ Steady-state O_2_ uptake during moderate-intensity exercise and ↑ time-to-task failure for 8.4 and 16.8 mmol NO_3_^−^
Wylie et al. (2013) [40]	14 (M)	Team sports athletes (VO_2max_ 52 ± 7 mL/kg/min)	4.1 mmol NO_3_^−^ twice daily for 2 consecutive days	Yo-Yo test (distance in m)	↑ Yo-Yo performance
Muggeridge et al. (2013) [20]	8 (M)	Kayak competitors (VO_2max_ 49 ± 6.1 mL/kg/min)	5.0 mmol NO_3_^−^	15 min rowing at 60 % W_max_ (power in W)	↓ VO_2_ ↔ Peak power or time trial performance
Christensen et al. (2013) [26]	10 (M)	Cyclists (VO_2max_ 72 ± 4 mL/kg/min)	5.0 mmol NO_3_^−^ 4 for 6 consecutive days	Repeated sprints (power in W) and time trial ≃1677 kJ (energy-expenditure-based time trial) (time in s and power in W)	↔ Vo_2_ kinetics ↔ Exercise economy ↔ Time trial performance
Hoon et al. (2014) [41]	28 (M)	Cyclists	4.1 mmol NO_3_^−^	4 min time trial (power in W)	↔ Time trial performance
Boorsma et al. (2014) [42]	8 (M)	Elite 1500 m runners (VO_2max_ 80 ± 5 mL/kg/min)	19.5 mmol NO_3_^−^	1500 m running time trial (time in s)	↔ VO_2peak_ ↔ Time trial performance
Martin et al. (2014) [43]	16 (9 M, 7 F)	Team sports athletes (VO_2max_ M: 57.4 ± 8 mL/kg/min; F: 47.2 ± 8 mL/kg/min)	4.83 mmol NO_3_^−^	8 s repeated sprints test on cyclic ergometer (number of sprints, work in kJ, power in W)	↔ Mean power output ↓ Number of sprints ↓ Total work
Peeling et al. (2015) [44]	6 (M)	National-level kayak competitors (VO_2peak_ 57.15 ± 2.8 mL/kg/min)	5.5 mmol NO_3_^−^	4 min maximal ergometer test (power in W and distance in m)	↓ VO_2_ ↑ Exercise economy ↑ Time trial performance
Porcelli et al. (2015) [21]	21 (M)	8 individuals with lower aerobic capacity (VO_2peak_ 28.2–44.1 mL/kg/min), 7 individuals with medium aerobic capacity (VO_2peak_: 45.5–57.1 mL/kg/min), and 6 individuals with high aerobic capacity (VO_2peak_: 63.9–81.7 mL/kg/min)	5.5 mmol NO_3_^−^ for 5 consecutive days	3 km running time trial	↑ Time trial performance for lower and medium aerobic capacity ↔ Time trial performance for high aerobic capacity
Wylie et al. (2015) [45]	10 (M)	Healthy and recreationally active	8.2 mmol NO_3_^−^ 3, 4 or 5 consecutive days	24 × 6 s sprints with 24 s rest; 7 × 30 s sprints with 240 s rest; 6 × 60 s sprints with 60 s rest (power in W)	↑ Power output for condition 1 ↔ Power output for conditions 2 and 3
McQuillan et al. (2017a) [27]	9 (M)	Cyclists (VO_2peak_: 68 ± 3 mL/kg/min)	9 mmol NO_3_^−^ 3 for 7 consecutive days	1 km time trial at fourth and seventh day of investigation and 4 km time trial at third in sixth day of investigation (time in s and power in W)	↔ Time trial ↔ Power output
McQuillan et al. (2017b) [28]	8 (M)	Cyclists (VO_2peak_ = 63 ± 4 mL/kg/min)	~4 mmol NO_3_^−^ for 8 consecutive days	4 km time trial (time in s in power in W)	↔ Time trial ↔ Power output
Christensen et al. (2017) [46]	17 (M)	8 recreationally active (VO_2max_ 46 ± 3 mL/kg/min) and 9 well-trained cyclists (VO_2max_: 64 ± 3 mL/kg/min)	9 mmol NO_3_^−^	Incremental test for cycling and arm cranking (power in W)	↔VO_2max_ ↑ Peak power for cycling ↔ Peak power for arm cranking
Nyakayiru et al. (2017a) [29]	17 (M)	Cyclists and triathlon competitors (65 ± 4 mL/kg/min, W_max_ 411 ± 35 W)	4 mmol NO_3_^−^ for 6 consecutive days	10 km time trial (time in s)	↔VO_2_ ↔ Time trial
Nyakayiru et al. (2017b) [24]	32 (M)	Football players	12.9 mmol NO_3_^−^ for 6 consecutive days	Yo-Yo test (distance in m)	↑ Covered distance
Vasconcellos et al. (2017) [47]	25 (14 M, 11 F)	Runners (M: VO_2peak_ 64.31 ± 4.71 mL/kg/min−1; F: VO_2peak_ 52.79 ± 4.57 mL/kg/min	9.92 NO_3_^−^ ± 1,97 mmol	High-intensity running (time in s)	↔ Time to fatigue ↔ VO_2max_ ↓ Blood glucose ↔ Systolic and diastolic blood pressures ↔ Serum cortisol, ↔ Blood lactate
Shannon et al. (2017) [48]	8 (M)	Runners in triathlon competitors (VO_2max_: 62.3 ± 8.1 mL/kg/min)	~12.5 mmol NO_3_^−^	Running for 1500 m and 10 000 m (time in s)	↔ Resting blood pressure ↑ Blood lactate for 1500 m time trial ↔ Blood lactate for 10,000 m time trial ↑ Time trial performance for 1500 m ↔ Time trial performance for 10,000 m
De Castro et al. (2018) [49]	14 (M)	Healthy and recreationally active runners (VO_2max_: 45.4 ± 5.9 mL/kg/min)	8.4 mmol NO_3_^−^ for 3 consecutive days	10 km running time trial (time in min and velocity in km/h)	↔ Time trial performance ↔ Mean velocity
Cuenca et al. (2018) [50]	15 (M)	Healthy and recreationally active	6.4 mmol NO_3_^−^	WAnT and CMJ (power in W, time to W_peak_)	↑ Peak and mean power output ↓ Time taken to reach W_peak_
Oskarsson et al. (2018) [51]	9 (M 7, F 2)	(M: VO_2max_ 59.0 ± 2.9 mL/kg/min; F: VO_2max_ 53.1 ± 11.4 mL/kg/min)	6.4 mmol NO_3_	1 km running time trial (time in s)	↔ Relative oxygen uptake, running economy, respiratory exchange ratio, HR, or RPE at submaximal intensities ↔ Performance, maximum HR, peak blood lactate concentration, or RPE during the maximal-intensity time trial
Jo et al. (2019) [52]	29 (M 15, F 14)	Healthy and recreationally active	8 mmol NO_3_^−^ for 15 consecutive days	8 km time trial (time in s, power in W, velocity in km/h)	Multiday NO_3_^−^ supplementation: ↑ Time trial performance ↑ Average power ↑ Velocity Single serving NO_3_^−^: ↔ Time trial performance ↔Average power ↔ Velocity
Rokkedal-Lausch et al. (2019) [53]	12 (M)	Cyclists (VO_2max_ 66.4 ± 5.3 mL/min/kg)	12.4 mmol NO_3_^−^ for 7 consecutive days	10 km time trial in normoxic and hypoxic conditions (time in s and power in W)	↑ Time trial performance in normoxic and hypoxic conditions ↔ HR ↔ Oxygen saturation ↔ Muscle oxygenation
Esen et al. (2019) [54]	10 (5 M, 5 F)	Swimmers with a minimum of 10 years training experience and minimum of 5 years competing experience	~800 mg NO_3_^−^ for 3 consecutive days	100 in 200 m swimming for time (time in s)	↔ Time trial performance for 100 and 200 m ↓ Systolic blood pressure
Wickham et al. (2019) [55]	12 (F)	Healthy and recreationally active (VO_2peak_: 40.7 ± 4.3 mL/kg/min)	Acute and chronic supplementation (~26 mmol) of NO_3_^−^ for either 1 or 8 consecutive days	10 min time trial at 50 and 70 % VO_2max_ (time in s)	↔ MVC voluntary activation ↔ Peak twitch torque, ↔ time to peak torque, ↔ half relaxation time ↔ Time trial performance ↔ VO_2_
Kent et al. (2019) [56]	12 (M)	Team sports athletes (VO_2peak_ 53.1 ± 8.7 mL/kg/min)	12.9 mmol NO_3_^−^	Four cycling sprints at sea and 3000 m altitude	↔ Peak and mean power
Mosher et al. (2019) [57]	11 (M)	Cyclists (VO_2max_: 60.8 ± 7.4 mL/kg/min)	12.8 mmol NO_3_^−^ for 3 consecutive days	40 km time trial (time in s)	↔ Time trial performance ↔ VO_2_ ↔ Blood lactate ↔ RPE
Ranchal-Sanchez et al. (2020) [58]	12 (M)	Healthy and recreationally active	6.4 mmol NO_3_^−^	Incremental test at 60, 70, and 80% maximal power for bench press and squat (number of repetitions, power in W, and velocity in m/s)	↑ NOR for 60 and 70% 1 RM ↔ NOR for 80% 1 RM ↑ NOR for squat ↔ NOR for bench press ↔ Power ↔ Velocity
López-Samanes et al. (2020) [59]	13 (M)	Professional tennis players	300 mg NO_3_^−^	Serving speed, CMJ, IHS, 5-0-5 agility test, and 10 m sprints test	↔ Serve velocity ↔ CMJ ↔ IHS ↔ 5-0-5 agility test ↔ Sprint performance
Liubertas et al. (2020) [60]	13 (M)	Healthy individuals	Acute and chronic intake (for 6 consecutive days) of 400 mg NO_3_^−^	Incremental cycling test first, third, and sixth day of investigation (power in W and VO_2max_)	↑ Peak power ↑ VO_2max_
Rodríguez-Fernández et al. (2020) [61]	18 (M)	Healthy and recreationally active	800 mg NO_3_^−^	Four sets of eight all-out half-squats with each set completed at different moment intertia (power in W)	↑ Mean and peak power output in the concentric and eccentric movement phases
Jonvik et al. (2021) [62]	15 (M)	Recreationally active	985 mg NO_3_^−^ for 6 consecutive days	CMJ, upper leg voluntary isometric (30° and 60° angle) and isokinetic contractions (60, 120, 180, and 300°s^−1^) and test of 30 reciprocal isokinetic voluntary contractions at 180°s^−1^	↔ CMJ ↔ Maximal isometric knee extensor strength and isokinetic knee extension power ↔ Muscular endurance
Dumar et al. (2021) [63]	10 (M)	National Collegiate Athletic Association sprinters	400 mg NO_3_^−^ 2 h prior to exercise	3 × 15 s WAnT with 2 min rest in the AM and PM (power in W and anaerobic capacity in Wkg^−1^)	NO_3_^−^ attenuated the decrease in AM exercise performance ↔ RPE ↓ HR
Marshall et al. (2021) [64]	22 (12 M, 10 W)	Healthy adults	~12.5 mmol NO_3_^−^ for 20 consecutive days	Harvard Step Test fitness at baseline (44 m altitude), 2350 m (day 9), 3400 m (day 12), and 4800 m (day 17)	NO_3_^−^ attenuated the decline in fitness scores with altitude ↑ HR recovery ↔ RPE ↔ High-altitude illness occurrence
Fowler et al. (2021) [65]	11 (M)	Healthy adults (VO_2max_: 41.1 ± 3.6 mL/kg/min)	∼9.2 mmol NO_3_^−^ for 5 consecutive days	Cycling exercise tolerance test in hot and dry conditions (35 °C, 28% relative humidity)	↔ Performance ↓ Arterial pressure ↔ Sweat rate ↔ Heart rate ↔ Oxygen consumption and carbon dioxide production ↔ Thermal sensation
Townsend et al. (2021) [66]	16 (M)	Division I baseball athletes	180 mg NO_3_^−^ daily for 11 weeks	1 RM bench press, WAnT, body composition analysis via a 4-compartment model	↔Perfromance (1 RM bench press and WAnT; observed trend for improved peak power in the WAnT) ↔ Body composition and muscle thickness ↔ HR and blood pressure

↑ = Significantly greater (*p* < 0.05) compared with placebo; ↔ = no significant change compared with placebo; ↓ = significantly lower (*p* < 0.05) compared with placebo; NO_3_^−^ = nitrates; BM = body mass; PCr = phosphocreatine; RPE = rating of perceived exertion; WAnT = Wingate anaerobic test; CMJ = counter movement jump; HR = heart rate; MVC = maximum voluntary strength; NOR = number of repetitions; 1 RM = repetition maximum; HIS = isometric grip strength. Potential reasons why some studies presented in Table 1 report a positive effect of NO_3_^−^ supplementation and others do not may result from different research methodological approaches, different testing, and supplementation protocols, potentially through the production of free radicals through NO reaction with O_2_, and differences between participants training status. The latter may be the most prominent reason why researchers report different findings, and many factors may contribute to this effect. The first is that intense exercise itself increases NOS activity [67], making top-tier athletes less dependent on the NO_3_^−^–NO_2_^−^–NO pathway of NO production and having higher baseline levels of NO_3_^−^ in comparison to less trained individuals [4]. Poveda et al. (1997) reported a 158% increase in baseline plasma NO_2_^−^ concentration in a sample of 10 well-trained runners and cyclists versus non-trained individuals [68]. Porcelli et al. (2015) reported that when VO_2max_ is low (<65 mL/kg/min), the effect of NO_3_^−^ on reducing oxygen consumption during exercise is most pronounced and can be as high as 10%. On the other hand, at higher VO_2max_ (>65 mL/kg/min), the effect of NO_3_^−^ is negligible [21]. It is worth noting that VO_2max_ is affected by both sports’ specificity as well as age. Certain elite-level athletes where NO_3_^−^ supplementation might be useful participating in sports requiring not only endurance, but also speed, strength, and anaerobic capacity might not have the same level of VO_2max_ as solely endurance athletes [69,70]. Additionally, in master athletes, there is a progressive decline of VO_2max_ with age [71]. Thus, a specific cut-off point at 65 mL/kg/min justifying the usage of NO_3_^−^ supplementation might or not might not be valid in all sports situations.
